# Supplementary material for: Netcombin: An algorithm for constructing optimal phylogenetic network from rooted triplets
Source: PLoS One. 2020 Sep 18;15(9):e0227842. doi: 10.1371/journal.pone.0227842 (PMC7500971; doi:10.1371/journal.pone.0227842)
Supplement: S4 File — (ZIP) [file pone.0227842.s004.zip › SequenceData-Triplet/sequenceData-10-20-40-Triplet2/data-(sample 10- length 500- num seq 40- m rate 10^-6)/6 seq.pdf]

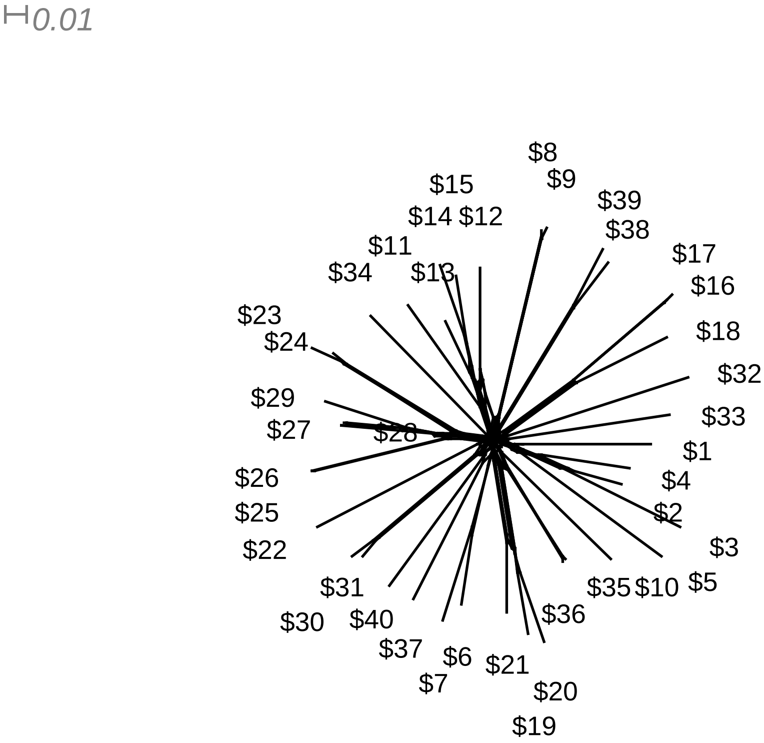
0.01
$8
$9
$15
$39
$14
$12
$38
$11
$17
$34
$13
$16
$23
$18
$24
$32
$29
$33
$27
$28
$1
$26
$4
$25
$2
$22
$3
$31
$35
$10
$5
$30
$40
$36
$37
$6
$21
$7
$20
$19
